# Determinants of Citation in Epidemiological Studies on Phthalates: A Citation Analysis

**DOI:** 10.1007/s11948-020-00260-y

**Published:** 2020-08-13

**Authors:** Miriam J. E. Urlings, Bram Duyx, Gerard M. H. Swaen, Lex M. Bouter, Maurice P. A. Zeegers

**Affiliations:** 1 Nutrition and Translational Research in Metabolism, School NUTRIM, Maastricht University, Maastricht, The Netherlands; 2 Care and Public Health Research Institute, School CAPHRI, Maastricht University, Maastricht, The Netherlands; 3 Department of Epidemiology and Data Science, Amsterdam University Medical Centers, Amsterdam, The Netherlands; 4 Department of Philosophy, Faculty of Humanities, Vrije Universiteit, Amsterdam, The Netherlands

**Keywords:** Citation bias, Network analysis, Questionable research practice, Phthalates, MEHP

## Abstract

Citing of previous publications is an important factor in knowledge development. Because of the great amount of publications available, only a selection of studies gets cited, for varying reasons. If the selection of citations is associated with study outcome this is called citation bias. We will study determinants of citation in a broader sense, including e.g. study design, journal impact factor or the funding source of the publication. As a case study we assess which factors drive citation in the human literature on phthalates, specifically the metabolite mono(2-ethylhexyl) phthalate (MEHP). A systematic literature search identified all relevant publications on human health effect of MEHP. Data on potential determinants of citation were extracted in duplo. Specialized software was used to create a citation network, including all potential citation pathways. Random effect logistic regression was used to assess whether these determinants influence the likelihood of citation. 112 Publications on MEHP were identified, with 5684 potential citation pathways of which 551 were actual citations. Reporting of a harmful point estimate, journal impact factor, authority of the author, a male corresponding author, research performed in North America and self-citation were positively associated with the likelihood of being cited. In the literature on MEHP, citation is mostly driven by a number of factors that are not related to study outcome. Although the identified determinants do not necessarily give strong indications of bias, it shows selective use of published literature for a variety of reasons.

## Introduction

The frequency of being cited is often used to assess the quality and scientific impact of a publication (Jannot et al. 2013). The rationale behind this is that high quality work will lead to more citations by peer scientists compared to low quality work (Bornmann and Daniel 2008). However, it can be questioned whether it is scientific merit only, that drives the number of citations, or other factors such as study outcome or the number of authors involved. It is known that only a few publications reach high citation counts whereas the majority of publications is cited only a few times at most (Ioannidis et al. 2016). Consequently, publications are forgotten over time whereas others remain to be cited, leading to potentially skewed knowledge development. Although we realise the amount of literature in most fields is too large to cite each available publication, thereby inevitable leading to selective citation, these citations should aim provide a balanced overview of the current level of knowledge in a field. Since scientific evidence is used as the basis for clinical and policy decisions, as well as for setting the future research agenda, selecting citations for the wrong reasons can have serious consequences.

Authors have different motivations to base the selection of their citations on, which can be either justified or unjustified. Study outcome is an unjustified determinant of citation, leading to citation bias (Duyx et al. 2017). Citation analyses have been performed in a large variety of research fields and with different methods. Citation analysis can be done in different contexts, for example within a demarcated research field (Greenberg 2009), within a specific journal (Nieminen et al. 2007; Kjaergard and Gluud 2002), or based on the individual publications included in a meta-analysis (Sawin and Robinson 2016). Also different methods can be distinguished to assess the relationship between study outcome and the chance of being cited. Ioannidis and Panagiotou (2011) compared the effect size of 35 highly cited publications with the effect sizes in meta-analyses on the same topics, whereas Greenberg (2009) compared the citation count towards positive and negative studies in the same field. Both studies found proof for the existence of citation bias. A recent systematic review and meta-analysis showed that citation bias is present in different research areas, with an average of approximately twice the number of citations for positive studies compared to negative studies (Duyx et al. 2017).

Contrary to study outcome, high quality of research is considered a justified determinant of citation. Most other determinants of citation will hold the middle between justified and unjustified determinants, e.g. journal impact factor, funding source or affiliation of the corresponding author. Journal impact factor is calculated on the basis of the total number of received citations by a journal in the previous 2 years (Moed et al. 2012; Garfield 2006). Although this cannot directly be translated to the chance of being cited for single publications, several studies did show a correlation between journal impact factor and the chance of citation (Bornmann and Daniel 2008). Also a correlation between study design and citation was reported, where case reports were least likely to receive high number of citations and meta-analysis were most likely to be highly cited (Patsopoulos et al. 2005). This seems legitimate, comparing the level of evidence prestented in meta-analyses compared to case reports. Industry funded research or industry affiliated researchers have been suggested to have a lower chance of being cited, as a result of lower perceived credibility of a for-profit organisation (Onodera and Yoshikane 2015; Conen et al. 2008). Furthermore, the gender of the author, number of references in a publication, number and type of affiliations included in a publication, the authors’ reputation and whether the title of the publication includes its conclusion or not have been studied in relation to citation, with varying results for different research fields (Bornmann and Daniel 2008; Onodera and Yoshikane 2015).

As a case study, we will study determinants of citation within the human literature on the harmful health effects of phthalates on humans. Phthalates are a used in plastic products, such as food packaging, toys and medical tubes and increase the flexibility of the plastic (National Research Council 2009). Phthalates are a group of different parent compounds such as diethyl phthalate (DEP), di-*n*-butyl phthalate (DBP), di-isonyl phthalate (DINP) and di-2-ethylhexyl phthalate (DEHP) (National Research Council 2009). They have been studied in relation of a variety of health outcome, such as human reproduction (Duty et al. 2005; Kay et al. 2013), obesity (Goodman et al. 2014) and ADHD in children (Kim et al. 2009). Due to the fast breakdown of phthalates in the body, phthalate exposure is mostly measured by means of metabolites in urine or blood. Since each parent compound breaks down into a number of metabolites, there is a great variety of phthalate metabolites that might be studied. In terms of citation analysis all these metabolites can be studied as separate subnetworks. In the current study we will focus on one metabolite: mono(2-ethylhexyl) phthalate (MEHP), which is one of the main metabolites of the parent compound DEHP and that can be detected in 75% of the US population (Duty et al. 2004; Mariana et al. 2016).

In the current citation analysis we combine all of the previously mentioned potential determinants of citation, to get a broad insight into the determinants of citation, specifically in the literature on phthalates and human health effects. Our main research question is therefore: Which determinants influence the likelihood of being cited in the scientific publications on harmful health effects of MEHP in humans?

## Methods

The design of this study was described in a study protocol, which was finalised prior to the data collection and data-analysis and was published online (https://bit.ly/2xhTrj1). The main steps of the citation network analysis will be described in the following paragraphs.

### Search Strategy and Article Selection

The network was composed on the basis of a systematic search in Web of Science–Core Collections performed in March 2018. A broad search strategy was applied to prevent missing important publications, namely “phthalate*” AND “human*” was searched in the title, abstract and key words. No limitations with regard to the health outcomes under study were applied. Identification of publications by checking the reference lists was not applied, since this would interfere with the research question. Reference checking would result in an overrepresentation of articles that are cited within the network, whereas articles that have been neglected by the network would still be missed.

The article selection was carried out in two phases. The first selection round was based on publication title, to limit the number of publications. The second selection round included reviewing abstracts, figures and tables, to finalize the article selection. The article selection was conducted individually by two researchers, MJEU and BD, followed by several consensus meetings.

### Data Extraction

All publications in the network were scrutinized for a number of characteristics that may be potential determinants of citation (see Table 1). Data extraction was performed independently by MJEU and BD. In all cases consensus was reached. Study outcome was scored in three ways; based on statistical significance, direction of the effect and authors’ conclusion. As the general hypothesis we take that MEHP leads to a harmful health effect. A publication was considered statistically significant when a *p* value lower than 0.05 was reported for the primary study outcome. In case multiple health outcomes were reported with *p* values both below and above 0.05, a publication was considered mixed. Odds ratios and relative risks higher than one were considered in line with the hypothesis that MEHP is harmful for human health, whereas point estimates between zero and one indicated absence of a harmful health effect. Finally, the study outcome was measured by studying the authors’ conclusion of the publication. This can be either in line with the hypothesis that a harmful health effect exists, which is mentioned as a positive study; or not in line with the hypothesis that a harmful health effect exists, which is mentioned as a negative study. The study designs presented in this network were observational studies (cohort, cross-sectional and case–control studies), systematic reviews and narrative reviews. The journal impact factor at the moment of publication was read from Clarivate Analytics’ Journal Citation Reports, via Web of Science.

**Table 1:** Overview of assessed determinants of citation and their operationalisation

Variable	Operationalisation
Statistical significance	*P* value for the primary study outcome related to MEHP and health (*p* < 0.05 is significant)
Direction of the effect	Protective or harmful effect according to the point estimate of the main study outcome, regardless of statistical significance (OR higher or lower than 1)
Authors’ conclusion	Study outcome as concluded by the author of the publication
Study design	Study design described in the publication (observational, experimental, review)
Sample size	Number of participants included in the publication
Title of the publication	Nature of the title of the publication: descriptive vs conclusive
Number of affiliations	Number of affiliations involved in the publication
Journal impact factor	Journal impact factor of the publication, measured at the time of citation, via Clarivate Analytics’ Journal Citation Reports
Funding source	Type of funding source of the publication (For profit vs not for profit)
Number of references	Number of references mentioned in the publication
Gender	Gender of the corresponding author
Affiliation	Affiliation of the corresponding author (university, industry, other)
Continent	Continent where the research was conducted
Authority of the author	Total number of citations received by each co-authors within the MEHP-network, upon the time of a new citation. Co-author with the highest authority score on a publication is tested as determinant of citation. This variable is not measured on the level of the publication and develops over time
Self-citation	One of the co-authors was involved in the cited and citing publication (yes or no). Self-citation is measured on the level of the citation instead of the publication

The determinant ‘authority of the author’ was measured on the publication’s level rather than citation level and developed over time. All co-authors of all publications received an ‘authority score’, which was the number of citations received within this MEHP network, during each year that the network was active. The authority of each publication was determined by the co-author with the highest authority score. We hypothesized that authors with a high authority would elevate the credibility of a publication and therefore would lead to a higher likelihood of being cited by future publications. In the analysis, we used the highest authority score on a publication, just before the citation under study. In this way the new citation does not impact the calculation of the authority score. Self-citation was defined as the situation in which at least one author was listed on both the cited and the citing publication in the MEHP-network. We analysed whether authors were more likely to cite themselves compared to others. For clarity purposes, all tested determinants and their operationalisation are displayed in Table 1.

### Statistical Analysis

Each publication in the network could take the role of citing and cited publication. First, we were interested in the effect of only the characteristics of the *cited* publication on the likelihood of being cited and therefore the unit of analysis was the potential citation path. A potential citation path existed between one publication and every other publication in the network that was available online or on paper at the moment of submission of the citing publication. In the data set, each row represented a potential citation path followed by an indication whether the potential citation path had actually been realized or not and the characteristics of the cited publication of that citation path. The overview of the actual citations was downloaded in April 2018.

A single publication normally cites multiple other publications, meaning that multiple citation pathways are leading to the same publication. These citation pathways are therefore not entirely independent. A multilevel approach was therefore required, in which the citation paths were nested under the citing publications. Random effect logistic regression was modelled to assess the effect of characteristics of the cited article on the likelihood of being cited.

Univariate analyses were performed to examine all potential determinants of citation described in Table 1. Second, all analyses were repeated while adjusting for study design, which was considered to be a proxy for study quality. Where initially a number of variables were measured on a continuous scale, these were transformed into categories for the analysis. This concerns the variables sample size, number of affiliations, journal impact factor, number of references and authority. Reason for this, was the very large spread in the data. Analysing these variables as continuous variables would lead to very small effect sizes that are difficult to interpret. Each of these variables was made into three categories, based on its tertiles.

Additionally, we assessed whether concordance between the characteristics of the cited and citing publication was a determinant of citation. A citation was scored as concordant in case it shared the same characteristic on the cited as well as the citing publication. In case the cited and citing publication scored differently on a variable, the citation scored negative on concordance. For example, with regard to study design: a citation was scored as concordant when both the cited and citing publications were cohort studies. In case the cited publication was a cohort study, while the citing publication was a case–control study, the citation was not scored as concordant. We hypothesized that authors would be more likely to cite publications that share characteristics with their own study. Via fixed effect logistic regression analysis we tested whether concordance between the cited and citing publication determined the likelihood of citation, for each of the assessed determinants. All statistical analyses were performed in Stata 13.

The outcomes of the logistic regression are reported as odds ratios. The odds ratio may overestimate the true relative risk in studies where the outcome is a common outcome (Cummings 2009). In our network, the overall chance of being cited is 9.6% (551 actual citations of 5684 potential citations). With this incidence, we consider ‘being cited’ relatively common, but we expect the overestimation of the true relative risk to be still acceptable. Ultimately, the odds ratio gives an accurate estimation of the direction of the effect; only the exact magnitude of the effect should be interpreted with some caution. For the sake of readability of the publication, we interpret these values as if they are relative risks and therefore speak about ‘the likelihood of being cited for negative studies compared to positive studies’.

## Results

The network consisted of 112 publications on human health effects of the metabolite MEHP, published between 2000 and 2018. The network consisted of 5684 potential citations of which 551 are actual citations, making the citation prevalence 9.6%. 37 publications in the network did not receive any citations. Six publications received more than twenty citations each, with a maximum of 33 citations. Four of these six highly cited publications were cross-sectional studies, all reporting non-significant findings with an effect size in line with the hypothesis that a harmful effect on health exists (Duty et al. 2003; Jonsson et al. 2005; Main et al. 2006; Hauser et al. 2006). The most cited publication was a cohort study, also reporting non-significant results with a point estimate in line with the hypothesis (Swan et al. 2005). Finally, a narrative review on human health and with an unclear conclusion on the health effect of MEHP received 27 citations within the network (Hauser and Calafat 2005).

The number of substances that were studied in addition to MEHP in one publication varied from one to 61, with a median of 8 substances. These substances were other phthalate metabolites and other endocrine disruptors such as bisphenol A and various heavy metals. The total number of health outcomes per publication ranged from one to 22, with a median of three. The majority of health outcomes were reproductive outcomes for men and women, such as semen quality, time to pregnancy and hormone fluctuation for a number of hormones. Furthermore, MEHP was studied in relation to metabolic outcomes (e.g. diabetes type 2, cancer, cardiovascular disease) and general health.

Table 2 gives an overview of the distribution of all tested determinants of citation over the 112 publications. The majority of publications reported a non-significant harmful health effect of MEHP. According to the authors’ conclusion, there seems to be no clear consensus on the topic. A large number of publications, 42 out of 112, did not draw a clear conclusion with regard to the health effect of MEHP. The number of publications concluding either a harmful or harmless effect of MEHP on human health, was almost similar. The majority of the publications describe empirical studies, with a great variation in sample size, ranging from 30 participants to 76 million participants, with a median of 240. The journal impact factor ranged from 0 to 9.8, with a median of 3.8.

**Table 2:** Distribution of article characteristics and determinants of citation of 112 human MEHP publications

Determinants	Categories	N publications	N citation paths (% actual citations)
Statistical significance	Non significant health effect	53	2705 (11%)
	Significant health effect	11	533 (10%)
	Both significant and non significant health effects	29	1350 (8%)
	No *p* values reported	19	1096 (7%)
Direction of the effect	No harmful health effect of MEHP	11	607 (4%)
	Harmful health effect of MEHP	42	2421 (12%)
	Mixed results	42	1731 (10%)
	No point estimate reported	17	925 (7%)
Authors’ conclusion	No harmful health effect of MEHP	30	1801 (12%)
	Harmful health effect of MEHP	35	1786 (10%)
	Mixed conclusion	5	156 (8%)
	Unclear conclusion	42	1941 (8%)
Study design	Cohort study	34	1168 (10%)
	Cross-sectional study	41	2596 (11%)
	Case–control study	10	946 (6%)
	Narrative review	10	657 (10%)
	Systematic review	6	273 (7%)
	Systematic review including meta-analysis	2	44 (5%)
Sample size	0–146 participants	26	1568 (11%)
	146–315 participants	31	1549 (10%)
	> 315 participants	37	1593 (8%)
Title of publication	Neutral title	93	4564 (9%)
	Title indicating conclusion	19	1120 (13%)
Number of affiliations	1–3 affiliations	48	2537 (9%)
	4–5 affiliations	33	1539 (9%)
	> 5 affiliations	30	1608 (11%)
Journal Impact Factor	< 3.2	38	1945 (7%)
	3.2–5.0	37	1895 (11%)
	> 5.0	37	1843 (11%)
Funding source	Not for profit funding	75	3588 (10%)
	For profit funding	1	48 (6%)
	Both for profit and not for profit funding	12	583 (9%)
	Funding not reported	22	1357 (8%)
	No funding applicable	2	108 (11%)
Number of references	0–40 references	37	1920 (9%)
	41–56 references	38	2015 (12%)
	> 56 references	37	1749 (8%)
Gender	Male	50	2112 (12%)
	Female	55	3063 (9%)
	Unknown	7	509 (7%)
Affiliation	University	88	4503 (10%)
	Government	18	996 (6%)
	Industry	1	48 (6%)
	Other	5	137 (15%)
Continent	North America	46	2609 (11%)
	Europe	30	1545 (9%)
	Asia	36	1530 (7%)

As displayed in Table 3, significant findings and a positive authors’ conclusion, in line with the hypothesis, were not found to be associated with a higher chance of being cited. The direction of the reported point estimate was associate with the chance of being cited: an effect size reflecting a harmful effect of MEHP was cited approximately 2.5 times more often compared to effect sizes reflecting no harmful health effect. The relationship between study outcome, either defined as statistical significance, direction of the effect or authors’ conclusion, could not be explained by the variation in the number of health outcomes studied in each publication.

**Table 3:** Crude and adjusted odds ratios (95% CI) for the likelihood of being cited

Determinant	Categories	Crude OR	Adjusted OR^a^
Significance	Non-significant results	1.00 (ref)	1.00 (ref)
	Significant results	0.96 (0.72–1.28)	0.98 (0.74–1.29)
	Mixed results	**0.77 (0.67–0.88)**	**0.75 (0.65–0.87)**
Direction of effect	No harmful effect	1.00 (ref)	1.00 (ref)
	Harmful effect	**2.79 (2.04–3.80)**	**2.46 (1.83–3.31)**
	Mixed results	**2.32 (1.59–3.38)**	**1.96 (1.41–2.74)**
Authors’ conclusion	No harmful health effect of MEHP	1.00 (ref)	1.00 (ref)
	Harmful health effect of MEHP	0.91 (0.78–1.06)	**0.79 (0.67–0.94)**
	Mixed conclusion	0.87 (0.59–1.27)	0.84 (0.58–1.23)
	Unclear conclusion	**0.69 (0.60–0.81)**	**0.62 (0.52–0.75)**
Study design	Narrative review	1.00 (ref)	1.00 (ref)
	Cross-sectional study	1.15 (0.89–1.50)	1.15 (0.89–1.50)
	Case–control study	0.70 (0.49–1.00)	0.70 (0.49–1.00)
	Cohort study	1.14 (0.85–1.54)	1.14 (0.85–1.54)
	Systematic review (incl. Meta-analyses)	0.76 (0.52–1.10)	0.76 (0.52–1.10)
Sample size^b^	0–146 participants	1.00 (ref)	1.00 (ref)
	146–315 participants	0.94 (0.78–1.14)	0.89 (0.74–1.07)
	> 315 participants	**0.77 (0.63–0.94)**	**0.69 (0.57–0.83)**
Title of publication	Neutral title	1.00 (ref)	1.00 (ref)
	Title indicating conclusion	**1.34 (1.13–1.58)**	**1.26 (1.07–1.49)**
Number of affiliations^b^	1–3 affiliations	1.00 (ref)	1.00 (ref)
	4–5 affiliations	1.19 (1.00–1.41)	1.11 (0.92–1.34)
	> 5 affiliations	1.17 (0.99–1.38)	1.06 (0.89–1.26)
Journal Impact Factor^b^	< 3.2	1.00 (ref)	1.00 (ref)
	3.2–5.0	**1.49 (1.28–1.74)**	**1.49 (1.27–1.75)**
	> 5.0	**1.51 (1.28–1.78)**	**1.51 (1.25–1.82)**
Funding source	Not for profit funding	1.00 (ref)	1.00 (ref)
	Both for profit and not for profit funding	0.90 (0.73–1.12)	0.93 (0.74–1.15)
	Funding not reported	**0.80 (0.69–0.93)**	**0.80 (0.67–0.95)**
	No funding applicable	1.22 (0.76–1.96)	**3.73 (1.43–9.72)**
Number of references^b^	0–40 references	1.00 (ref)	1.00 (ref)
	41–56 references	**1.31 (1.09–1.56)**	**1.28 (1.07–1.52)**
	> 56 references	0.92 (0.77–1.11)	**0.79 (0.64–0.96)**
Gender^c^	Male	1.00 (ref)	1.00 (ref)
	Female	**0.78 (0.66–0.92)**	**0.71 (0.60–0.83)**
Affiliation	University	1.00 (ref)	1.00 (ref)
	Government	**0.64 (0.53–0.76)**	**0.66 (0.54–0.81)**
	Industry	0.74 (0.33–1.67)	1.07 (0.40–2.82)
	Other	1.34 (0.89–2.03)	1.25 (0.81–1.93)
Continent	North America	1.00 (ref)	1.00 (ref)
	Europe	**0.83 (0.71–0.97)**	**0.83 (0.70–0.98)**
	Asia	**0.66 (0.55–0.79)**	**0.66 (0.56–0.78)**
Authority^b^	0–6	1.00 (ref)	1.00 (ref)
	7–39	**1.49 (1.24–1.79)**	**1.43 (1.17–1.75)**
	> 40	**1.66 (1.36–2.03)**	**1.64 (1.33–2.02)**

^a^Model adjusted for study design, as proxy for study quality

^b^Variables are categorised based on tertiles

^c^Publications from corresponding author with unknown gender were not included in this analysis

Bold figures are statistically significant findings (*p* < 0.05)

Other determinants significantly associated with a higher chance of being cited, although with limited effect sizes, were: journal impact factor, authority of the author, male corresponding author, being located in North America and stating the conclusion in the title of the publication. Content related variables, such as study design and sample size, did not show clear or conclusive association with the chance of being cited.

As shown in Table 4, where we assessed concordance between the cited and citing publication, the impact of the characteristics of the *citing* publication on the chance of citation appeared limited. With regard to study design, sample size, gender and continent a small effect was found that authors were more likely to cite publications that were similar to their own characteristics. The only factor that was of great influence is self-citation; authors were three times more likely to cite their own work compared to that of others.

**Table 4:** Concordance analsyses of the characteristics of the cited and citing publications

Determinant	Crude OR	Adjusted OR^a^
Significance	0.78 (0.54–1.13)	0.76 (0.52–1.11)
Direction of the effect	0.94 (0.77–1.14)	0.88 (0.72–1.07)
Authors’ conclusion	0.89 (0.73–1.09)	0.88 (0.72–1.07)
Study design	**1.75 (1.41–2.17)**	**1.75 (1.41–2.17)**
Sample size	**1.53 (1.21–1.93)**	**1.53 (1.21–1.93)**
Title of publication	0.92 (0.76–1.10)	0.96 (0.79–1.16)
Number of affiliation	1.18 (0.98–1.41)	1.16 (0.97–1.40)
Journal impact factor	0.80 (0.64–1.01)	0.82 (0.65–1.03)
Funding source	1.02 (0.85–1.21)	1.01 (0.84–1.21)
Number of references	1.08 (0.90–1.30)	1.08 (0.90–1.30)
Gender	**1.25 (1.04–1.50)**	**1.24 (1.03–1.50)**
Affiliation	1.06 (0.88–1.28)	1.03 (0.85–1.24)
Continent	**1.40 (1.17–1.68)**	**1.40 (1.17–1.68)**
Self-citation	**3.22 (2.52–4.11)**	**3.16 (2.46–4.06)**

^a^Adjusted for study design

Bold figures are statistically significant findings (*p* < 0.05)

## Discussion

Our study shows that citation bias is only partly present in the literature on MEHP. Reporting of significant results and the authors’ conclusion do not influence the likelihood of being cited. However, a point estimate indicating a harmful effect of MEHP does increase the chance of being cited by 2.5 times. This effect cannot be explained by the study design or the number of health outcomes reported in each publication. Since the aim of the study was also to create a broader view on determinants of citation, we did identify other variables that significantly influence the chance of being cited in MEHP literature. These were mostly factors that related to the author of the cited publication, such as the authority and gender of the corresponding author as well as self-citation. Additionally, article characteristics that do not relate to the content of the publication, e.g. journal impact factor and the title of the publication, were significantly associated with the chance of being cited. Factors that relate to the content of the publication, such as the study design, were not associated with the chance of being cited. Most of the identified odds ratios were not very extensive in terms of magnitude, except self-citation and reporting of a harmful point estimate, which might be good news for knowledge development. Nevertheless, knowing that MEHP and phthalates as a whole are a politically sensitive topic, it might need awareness that relatively much attention is going to rather small studies, studies performed in North-America, and with relatively high impact of the authority of authors and high impact factor journals. On the other hand, looking at the 37 publications that have received no citations within this network up to April 2018, high-quality research might have been neglected.

Also we did some rather remarkable findings, that might demonstrate the large impact of the low number of highly cited publications. An example of this, is the positive association between studies that did not require funding and the likelihood of citation. This effects seems to be related to study design, since it appears after adjusting for this variable. A potential explanation for this would be, that narrative reviews are often publications that do not require funding. One of the highly cited papers in this networks is actually a narrative review, potentially impacting the identified association. Another remarkable finding is the negative association between higher sample size and likelihood of citation, which is opposite from what is expected. Also here, there might be a heavy impact of the highly cited studies with low number of participants.

Apart from the described outcomes of the citation analysis, we did observe some interesting network-specific points, while mapping the literature on MEHP and health and performing this citation analysis. This included the large number of health outcomes that were studied in relation to MEHP and other phthalates. This variation created several kind of subnetworks within the network, with each a rather limited number of publications. Unfortunately, these subnetworks were too small in number of publications in order to perform separate analysis.

Recently, the literature on harmful health effects of phthalates has been the subject of a study on outcome reporting bias (Swaen et al. 2016). Outcome reporting bias refers to the situation where only a selection of the originally measured outcomes, most likely the positive findings, are reported in the publication (Dwan et al. 2008). To prevent outcome reporting bias, research should be based on a preregistered research protocol. This study protocol is needed to distinguish between confirmatory testing and exploratory analyses, which is crucial in interpreting statistical results (Veldkamp et al. 2018). Previous research by Swaen et al. (2016) tested the occurrence of outcome reporting bias in the literature on phthalates, by comparing the methods and results of the publications to the planned analyses as laid down in a research protocol. They found that only a very small proportion of the phthalate publications were based on a research protocol. In addition, the research protocols that were used, lacked details to reproduce the study, removing the possibility to verify whether selective reporting of outcomes was present (Swaen et al. 2016). Just like citation bias, outcome reporting bias is a questionable research practice (QRP). QRPs are more subtle misbehaviors, compared to fraud and fabrication of data, yet difficult to recognise and therefore a serious threat to the validity and credibility of science (Fanelli 2011). Both outcome reporting bias and citation bias relate to selective communication of information and thereby hinder correct knowledge development (Song et al. 2010). In the literature on phthalate these biases are especially relevant, because phthalates are associated to many different health outcomes, which makes it a field that is prone to false positive findings.

Our study had several limitations. Because MEHP is often studied together with other substances and in relation to a large variation of health outcomes, it is difficult to demarcate a network of publications that should refer to each other. Since we did not score the location or content of the citations, it is possible that a part of the citations did not refer to the effect of MEHP, but to some of the other substances that were included in the same publication. Additionally, we are aware of the fact that we have tested a high number of associations, without adjusting for multiple testing. Since most tested hypotheses were independent from each other, the risk of a false positive finding for each test was not interlinked. Adjusting for multiple testing would in that case not increase the accuracy of the research. Only for study outcome, which was operationalized in three ways, multiple testing could lead to misinterpretation of the results. However, these results did not change when we reanalysed the data with a Bonferroni correction.

In conclusion, we can state that the amount of citation bias is limited in the human literature on the harmful health effects of MEHP. However, the chance of citation was significantly associated with the reporting of a harmful point estimate for the relation between MEHP and health, the journal impact factor, male gender, authority of the author, geographic location, the title of the publication and self-citation.

## Data Availability

The predefined study protocol, including code book and statistical analysis plan, and data set are available on the data repository dataverse: https://bit.ly/2xhTrj1.
